# Association of dialysis-related amyloidosis with lower quality of life in patients undergoing hemodialysis for more than 10 years: The Kyushu Dialysis-Related Amyloidosis Study

**DOI:** 10.1371/journal.pone.0256421

**Published:** 2021-08-24

**Authors:** Kazuhiko Tsuruya, Hisatomi Arima, Kunitoshi Iseki, Hideki Hirakata, The Kyushu Dialysis-Related Amyloidosis Study Group

**Affiliations:** 1 Department of Nephrology, Nara Medical University, Kashihara, Japan; 2 Department of Integrated Therapy for Chronic Kidney Disease, Graduate School of Medical Sciences, Kyushu University, Fukuoka, Japan; 3 Department of Preventive Medicine and Public Health, Fukuoka University, Fukuoka, Japan; 4 Clinical Research Support Center, Nakamura Clinic, Urasoe, Japan; 5 Fukuoka Renal Clinic, Fukuoka, Japan; Medical University of Gdansk, POLAND

## Abstract

**Background:**

Dialysis-related amyloidosis (DRA) commonly develops in patients undergoing long-term dialysis and can lead to a decline in activities of daily living and quality of life (QOL), mainly owing to orthopedic complications.

**Methods:**

First, we determined utility scores of the EuroQol 5-Dimensions 3-Levels (EQ-5D-3L) questionnaire in 1,323 patients with DRA who had undergone dialysis for more than 10 years and compared the score between those with and without DRA. Second, a 2-year follow-up was also performed, in which patients were divided into three groups: those complicated by DRA from the beginning, those with newly developed DRA within the 2-year period, and those not complicated by DRA throughout the survey period; changes in the EQ-5D-3L utility score were compared. In the group already complicated by DRA at the survey baseline, changes in the EQ-5D-3L utility score were compared according to the dialysis treatment method.

**Results:**

A total of 1,314 and 931 patients were included in the first and second studies, respectively. EQ-5D-3L utility scores among patients diagnosed with DRA were significantly lower than scores in those not diagnosed with DRA. The reduction in the EQ-5D-3L utility score over the 2-year follow-up was significantly greater in patients newly complicated by DRA during the follow-up period after enrollment but not in those complicated by DRA from the beginning, as compared with patients not complicated by DRA throughout the survey period. The reduction in utility score tended to be lower in patients routinely treated with a β2-microglobulin adsorption column than in untreated patients with DRA.

**Conclusion:**

Complication by DRA in patients undergoing long-term hemodialysis was significantly associated with a decline in QOL.

## Introduction

Dialysis-related amyloidosis (DRA) was initially reported by Warren and Otieno in 1975, in which carpal tunnel syndrome frequently developed among patients undergoing long-term dialysis [1]. Gejyo et al. [2] subsequently clarified in 1985 that DRA develops owing to deposition of β2-microglobulin. The association between the time-course frequency of complication by DRA and the β2-microglobulin removal efficiency of the dialysis membrane was investigated. The findings showed that the DRA complication rate was suppressed through the application of high-performance membranes. However, complete prevention of complication by DRA is difficult and there is concern that the number of such patients worldwide will increase because an increase in the population requiring dialysis is anticipated, especially in Asia. Moreover, there are many unclear points with respect to accurately determining the prevalence and actual state of DRA because there are no clear diagnostic criteria. There are currently two guidelines concerning DRA in Japan [3,4] in which the importance of diagnosis and treatment is emphasized.

Activities of daily living (ADL) and quality of life (QOL) in patients with DRA generally decline owing to orthopedic complications [5,6]. A survey of the actual state of DRA, performed by the Research Committee of Amyloidosis of the Ministry of Health, Labour and Welfare of Japan, showed the possibility that arthralgia is involved in gait disorders [7]. Gait disorders may adversely affect the life prognosis, as indicated by the pathological concepts of sarcopenia and frailty [8,9]. Bone lesions in DRA are not described in the Kidney Disease: Improving Global Outcomes (KDIGO) Clinical Practice Guideline on Chronic Kidney Disease-Mineral and Bone Disorder (CKD-MBD) [10], but the possibility of an association of DRA with the risk of femoral neck fracture was discussed in the KDIGO-CKD-MBD-Summit (Tokyo) in 2018 [11]. Because femoral neck fracture is a complication with a poor outcome [12], early therapeutic intervention is important.

A survey of the actual state of another DRA complication, carpal tunnel syndrome, has been reported in a nationwide survey of dialysis patients in Japan [13,14]. Repeated previous surveys have clarified that the prevalence of carpal tunnel syndrome has recently tended to decrease, and this is considered to be owing to improvement in the quality of dialysis treatment. This was further supported by results of a single-center study performed in Germany [15].

In the Dialysis Outcomes and Practice Patterns Study (DOPPS) using the dialysis-related amyloidosis questionnaire (DRAQ) designed by Chertow et al. [7], elevated DRAQ score was associated with a decline in QOL [16]. According to the amyloid score designed by Hoshino et al. [17], an elevated score is also associated with the physical functioning scale of the Short-Form (SF) 36.

Based on this background, we diagnosed DRA according to the diagnostic criteria of DRA standardized by the Research Committee of Amyloidosis of the Ministry of Health, Labour and Welfare and attempted to quantitatively evaluate QOL using the EuroQol 5-Dimensions 3-Levels (EQ-5D-3L) questionnaire, which is a standardized QOL evaluation system that is used worldwide [18]. Herein, we report our investigation of a cohort involving 1,314 patients undergoing hemodialysis for more than 10 years at 72 facilities in Kyushu and Okinawa, Japan. The presence or absence of complication by DRA was judged based on the clinical diagnostic criteria of DRA prepared by the Research Committee of Amyloidosis of the Ministry of Health, Labour and Welfare, and QOL was evaluated using the EQ-5D-3L. In addition, at the 2-year follow-up, we investigated the association of three conditions (already complicated by DRA at baseline, newly complicated during the follow-up period, and not complicated by DRA) with QOL. Furthermore, we evaluated the association between differences in the blood purification method and the time-course of changes in QOL, to investigate the possible use of a standardized DRA diagnosis-based treatment strategy in the maintenance or improvement of QOL.

## Methods

### Participants

**Fig 1:**
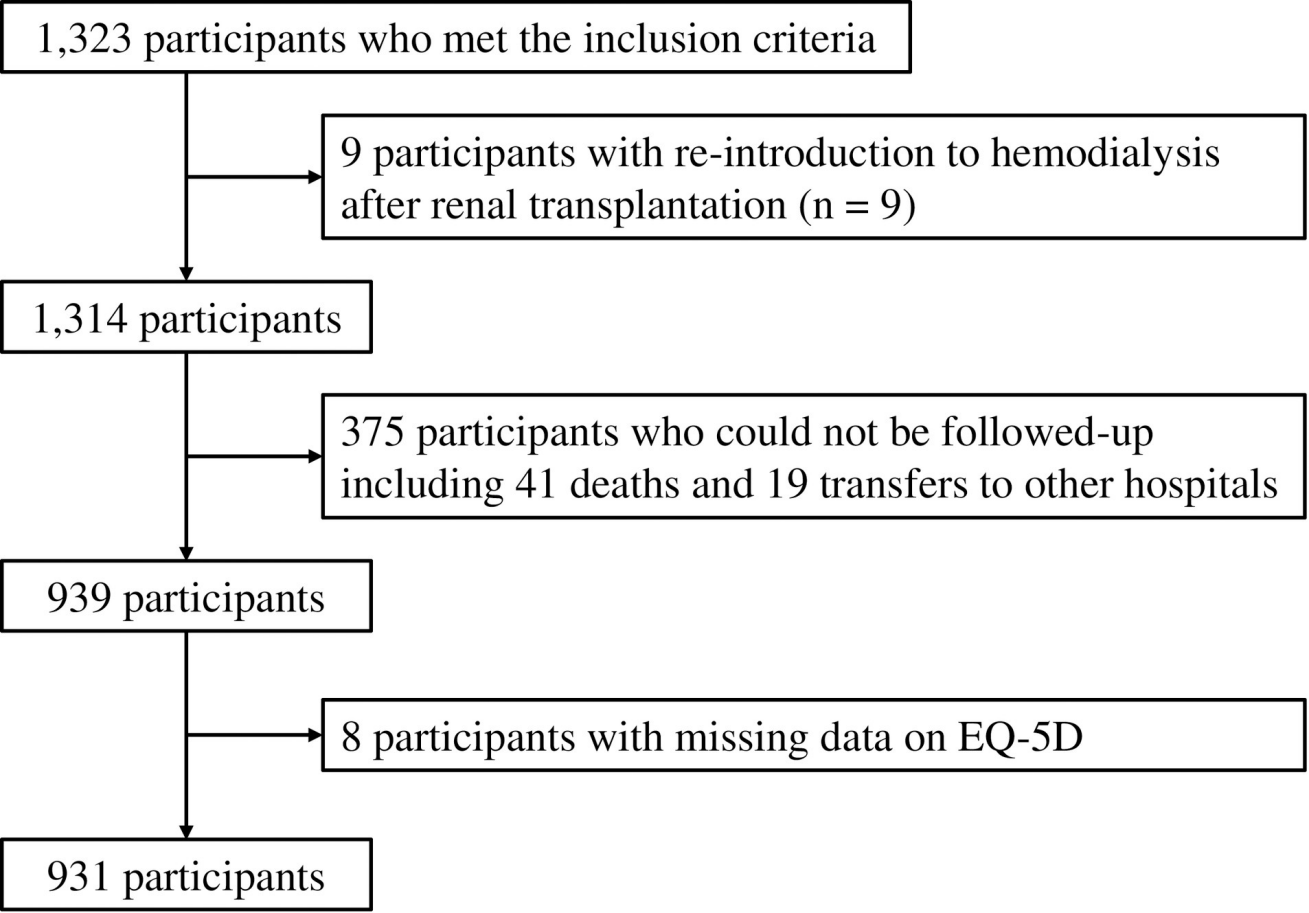
Patient flow diagram.

The first study: We conducted a questionnaire survey of 1,323 patients who had been undergoing chronic dialysis for more than 10 years at 72 hemodialysis facilities in Kyushu and Okinawa and who gave their consent to participate in the study. We excluded nine patients in whom dialysis was reintroduced after kidney transplantation; the remaining 1,314 patients were included in the analysis (**Fig 1**).

The second study: Of the patients who participated in the first study, those who gave their consent to repeat the survey after 2 years were included. Participants comprised 931 patients who underwent treatment at the same facility as that in the first study 2 years earlier and completed the EQ-5D-3L. After obtaining their consent, 41 patients were unable to be surveyed because they had died, and 19 patients could not be surveyed because they had been transferred to another hospital (**Fig 1**).

### Ethical approval

This study was conducted in accordance with the principles of the Declaration of Helsinki and was approved by Hattori Clinic Ethics Review Board (approval ID: the first study, F1307-T05; the second study, F1412-T05). The ethics committee of all participating institutions waived the requirement for written informed consent because of the retrospective nature of the present study. We obtained written informed consent from each patient.

### Evaluation of QOL and survey items

QOL was surveyed using the EQ-5D-3L. In the EQ-5D-3L, patients selected a response from among three options to the following five questions: (1) do you have any problems with mobility? (2) do you have any problems with self-care? (3) do you have any problems with usual activities? (4) do you have any problems with pain or discomfort? (5) do you have any problems with anxiety or depression?

A unique health state is defined by combining one level from each of the five dimensions. A total of 243 (3^5^) possible health states is defined in this way. Each state is referred to in terms of a 5-digit code. By adding unconsciousness and death to the 243 conditions, a total of 245 health conditions can be presented as a utility score: (1) mobility, (2) self-care, (3) usual activities, (4) pain/discomfort, (5) anxiety/depression (**S1 Table**) [19]. In the first study, the outcome was high EQ-5D-3L utility score, which was defined as a utility score of more than the median value. In the second study, the outcomes were a change in the EQ-5D-3L utility score (EQ-5D-3L utility score after 2-year follow-up − EQ-5D-3L utility score at baseline) and a decline in EQ-5D-3L utility score (change <0, i.e., any decline in the utility score).

In the survey on DRA, the presence or absence of the following eight conditions was confirmed according to the diagnostic criteria established by the Research Committee of Amyloidosis of the Ministry of Health, Labour and Welfare: (1) polyarthralgia, (2) past medical history of carpal tunnel release, (3) bone cyst, (4) destructive spondyloarthropathy or thickening of the cervical ligament, (5) snapping finger, (6) femoral neck fracture, (7) ischemic colitis, and (8) subcutaneous mass; the five major criteria include (1) to (5), and three minor criteria comprise (6) to (8). Patients with two or more major criteria were diagnosed with DRA.

The following items were recorded in the medical records: Patient background (age sex; dialysis vintage; primary disease past medical histories of myocardial infarction, cerebral hemorrhage, cerebral infarction, and amputation), physical findings (height, pre-dialysis body weight, post-dialysis body weight, body mass index [BMI]), blood data (Kt/V, creatinine generation rate, protein catabolism rate, geriatric nutritional risk index [GNRI], pre-dialysis β2-microglobulin concentration, white blood cell count, platelet count, albumin, hemoglobin, ferritin, transferrin saturation, serum iron, C-reactive protein [CRP], triglycerides, total cholesterol, low-density lipoprotein cholesterol, and high-density lipoprotein cholesterol), chest radiography (cardiothoracic ratio), blood pressure (pre-dialysis blood pressure at the beginning of the week), dialysis method (hemodialysis, hemofiltration, hemodiafiltration [dilution method, replacement fluid volume], concomitant use of a β2-microglobulin adsorber, Lixelle^®^, dialysis membrane, hemodiafilter, dialysis time, blood flow rate, dialysate purification [endotoxin level, bacterial count], and drug administration [erythropoiesis stimulating agent preparation, iron agent, benzodiazepines, antidepressants].

The following items were investigated in a survey concerning facilities: number of physicians (internists, surgeons), nurses, clinical engineers, case workers, dietitians (full-time, part time), and dialysis patients (daytime dialysis, nighttime dialysis, peritoneal dialysis by hospital visit or admission, and home dialysis), time of initiation of online hemodiafiltration treatment.

### Statistical analyses

Data are expressed as mean ± standard deviation (SD), median (interquartile range), or n (percentage), as appropriate. The differences among groups were compared using the Kruskal–Wallis test for continuous variables and chi-squared test for categorical variables. In the first study, factors associated with high EQ-5D-3L utility scores were investigated using multivariable logistic regression models with/without the number of main symptoms of DRA as a covariate. In the second study, we investigated the effects of DRA status (Group 1: participants complicated by DRA from the beginning, Group 2: those with newly developed DRA within the 2-year period, and Group 3: those not complicated by DRA throughout the survey period) on the outcome of decline in EQ-5D-3L utility scores after 2 years using univariable and multivariable logistic regression models. We also investigated the effects of DRA status (Groups 1 to 3) on the outcome of changes in EQ-5D-3L utility scores during the 2-year follow-up period were investigated using one-way analysis of variance (ANOVA) for crude analysis and analysis of covariance (ANCOVA) for multivariable analysis. In the multivariable analysis (both logistic regression analysis and ANCOVA), age, age at hemodialysis onset, sex, diabetic nephropathy, dialysis vintage, history of myocardial infarction, ischemic stroke, intracerebral hemorrhage, amputation, predialysis BMI, Kt/V, geriatric nutritional risk index, hemoglobin, CRP, and use of benzodiazepine agents were adjusted in Model 1; new-onset myocardial infarction, ischemic stroke, intracerebral hemorrhage, and amputation during follow-up were further adjusted in Model 2; all factors in Model 2 except for Kt/V and CRP were adjusted in Model 3 (because of missing values in these variables among approximately 20% of participants). A two-tailed *P*-value < 0.05 was considered statistically significant for all analyses.

## Results

### The first study

A total of 1,323 patients were enrolled initially and after excluding nine patients in whom dialysis was reintroduced after kidney transplantation, the remaining 1,314 patients were included in the first study. Patients’ characteristics and blood data at baseline are shown in **S2 Table**. Participants’ mean age was 64.7 years and the median dialysis vintage was 17 years. The mean β2-microglobulin level was 28.8 mg/L, which was below the 30.0 mg/L level specified as a life prognostic factor in the Japanese Society for Dialysis Therapy guidelines [20]. GNRI was 91.1, suggesting a slight nutritional disturbance. The dialysis efficiency was controlled at Kt/V 1.75. No noteworthy findings were identified for the other test values. Of the eight survey items regarding symptoms of DRA, the proportion of patients with polyarthralgia was 27% (250 patients), which was the highest proportion; this was followed by carpal tunnel release in 21% (191 patients) and snapping finger in 15% (140 patients). Patients without any conditions suggestive of DRA accounted for 58% (540 patients) of the population. Twenty-one percent of respondents (192 patients) had two or more major symptoms and were clinically diagnosed with DRA.

**Fig 2:**
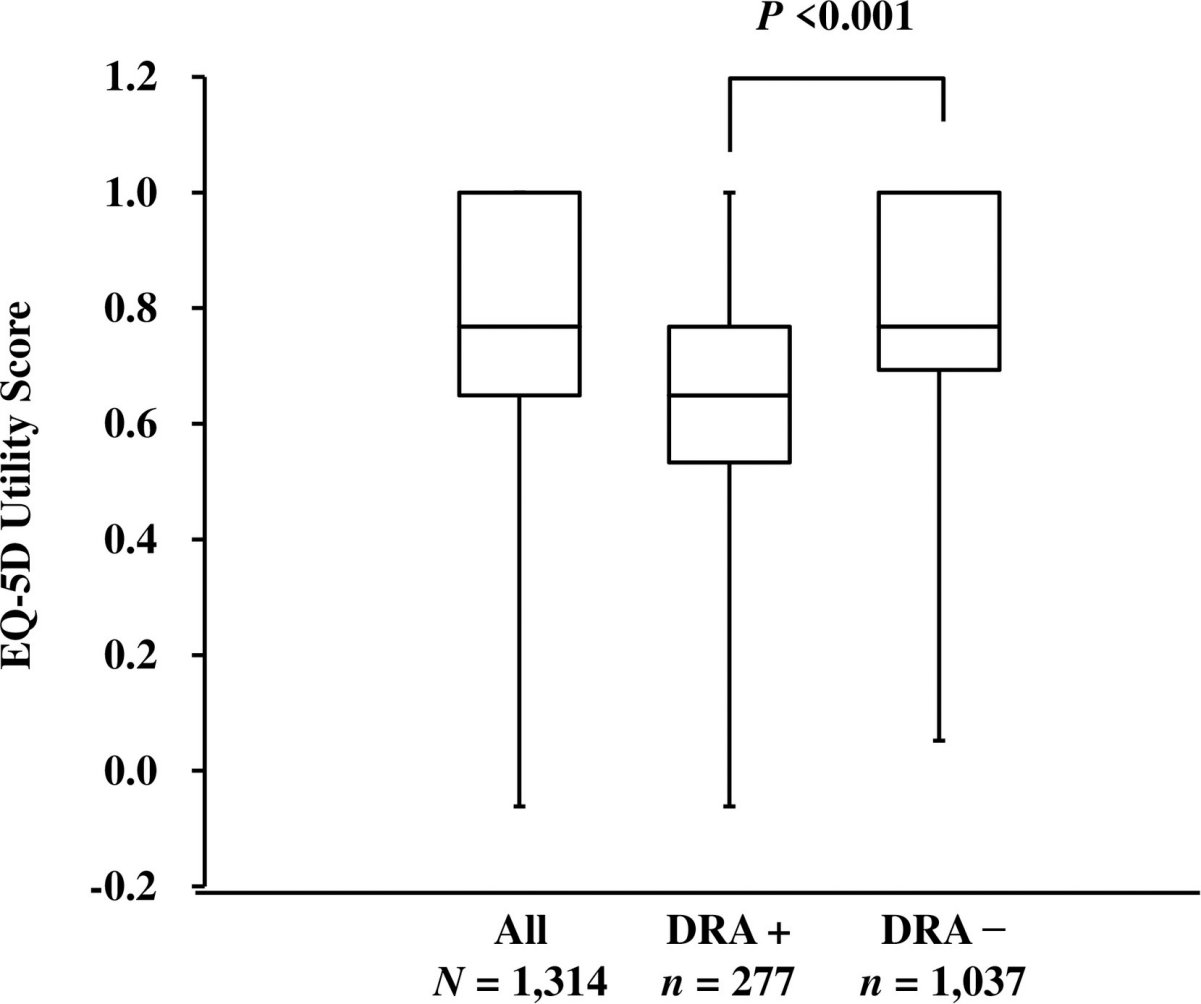
EQ-5D-3L utility scores according to the presence of DRA at baseline. EQ-5D-3L utility scores are shown for all patients and for those with and without DRA. Graphs show the median (center line of box), 1st quartile (lower edge of box), 3rd quartile (upper edge of box), maximum (upper end of range line), and minimum (lower end of range line) of utility scores. Abbreviations: DRA, dialysis-related amyloidosis; EQ-5D-3L, EuroQol 5-Dimensions 3-Levels.

In response to questions of the EQ-5D-3L, the highest proportion of patients reported some problems with “pain/discomfort”, accounting for 55% of the population, followed by “mobility” accounting for 36% and “usual activities” accounting for 33% (**S1 Table**) of participants. When the utility score was determined based upon these answers, the median of the 1,314 patients was 0.768, showing a bimodal distribution (**S1 Fig**). With respect to the presence or absence of clinically diagnosed DRA, the utility score was 0.649 in patients with DRA and 0.768 in patients without DRA, showing that the score was significantly lower in patients with DRA (**Fig 2**).

To investigate factors associated with the EQ-5D-3L utility score, we performed multivariable logistic analysis of the survey items and utility score (**S3 Table**). Male sex and higher GNRI were significantly associated with higher utility score. In contrast, age, dialysis vintage, diabetic nephropathy, cardiovascular complications (complicated by one or more past medical histories of myocardial infraction, cerebral hemorrhage, cerebral infarction, and amputation), BMI, and use of benzodiazepines were significantly associated with lower utility score. Clinically diagnosed DRA and complication by three or more major symptoms was also significantly associated with lower utility score, and its relative risk was similar to that of cardiovascular complications.

### The second study

EQ-5D-3L was reevaluated after follow-up for 2.0 ± 0.4 years after the first survey of the 931 patients. Among these, 192 (20%) were already complicated by DRA at baseline (Group 1), 44 (5%) newly developed DRA during the follow-up period (Group 2), and 695 (75%) were not complicated by DRA (Group 3), as shown in **Table 1**. While the dialysis vintage was significantly shorter, the pre-dialysis β2-microglobulin level was significantly higher in Group 3 (**Table 1**).

**Table 1:** Baseline characteristics and laboratory data according to DRA status after follow-up periods.

	Group 1 (*n* = 192)	Group 2 (*n* = 44)	Group 3 (*n* = 695)	*P* value			
				ANOVA	G1 vs. G2	G1 vs. G3	G2 vs. G3
Age, years	64.7 ± 8.4	63.7 ± 10.5	63.2 ± 10.6	0.330	0.810	0.147	0.617
Age at HD initiation, years	38.5 ± 11.2	40.9 ± 12.4	46.3 ± 12.5	<0.001	0.096	<0.001	0.006
Male, n (%)	93 (48%)	21 (48%)	394 (57%)	0.082	0.932	0.042	0.245
Diabetic kidney disease, n (%)	8 (4%)	3 (7%)	78 (11%)	0.011	0.452	0.003	0.364
Dialysis vintage, years	27 (21–32)	23 (17–28)	15 (12–20)	<0.0001	0.003	<0.001	<0.001
Previous history, n (%)							
Acute myocardial infarction	16 (8%)	2 (5%)	33 (5%)	0.151	0.393	0.056	0.948
Brain hemorrhage	8 (4%)	3 (7%)	22 (3%)	0.391	0.452	0.499	0.195
Brain infarction	18 (9%)	5 (11%)	65 (9%)	0.909	0.688	0.994	0.666
Amputation of extremities	0 (0%)	0 (0%)	6 (1%)	−	0.688	0.196	0.535
Body height, cm	157.6 ± 9.0	157.9 ± 9.2	160.1 ± 9.0	0.002	0.784	0.001	0.158
Pre-dialysis body weight, kg	54.1 ± 10.4	53.5 ± 11.1	56.4 ± 10.9	0.005	0.732	0.004	0.081
Post-dialysis body weight, kg	51.9 ± 10.1	51.3 ± 11.0	54.0 ± 10.5	0.011	0.737	0.007	0.105
Pre-dialysis BMI, kg/m^2^	21.6 ± 2.9	21.3 ± 3.3	21.9 ± 3.2	0.178	0.587	0.127	0.227
Post-dialysis BMI, kg/m^2^	21.6 ± 2.9	21.3 ± 3.3	21.9 ± 3.2	0.178	0.587	0.127	0.227
Pre-dialysis SBP, mmHg	142.0 ± 21.4	136.2 ± 21.3	146.3 ± 22.3	0.004	0.063	0.062	0.003
Pre-dialysis DBP, mmHg	73.4 ± 12.7	72.7 ± 13.1	77.0 ± 13.4	0.002	0.670	0.002	0.042
Cardiothoracic ratio, %	50.9 ± 4.8	50.0 ± 4.0	50.1 ± 5.2	0.090	0.256	0.030	0.980
EQ-5D-3L utility score	0.693 (0.587–0.768)	0.768 (0.596–1.00)	0.786 (0.705–1.00)	<0.0001	0.056	<0.001	0.003
Kt/V	1.74 ± 0.38	1.80 ± 0.37	1.79 ± 0.49	0.735	0.447	0.679	0.534
CGR, %	95.7 ± 34	106.1 ± 35.2	114.9 ± 33.4	<0.0001	0.013	<0.0001	0.409
nPCR, g/kg/day	0.96 ± 0.20	0.94 ± 0.17	0.95 ± 0.21	0.696	0.429	0.542	0.602
GNRI	95.2 ± 7.8	94.1 ± 8.7	96.8 ± 7.9	0.006	0.399	0.008	0.037
β2-MG, mg/L	27.0 ± 9.4	27.3 ± 4.5	29.3 ± 5.9	<0.0001	0.199	<0.001	0.029
Hemoglobin, g/dL	10.9 ± 1.2	10.8 ± 1.0	11.0 ± 1.1	0.661	0.658	0.577	0.435
White blood cells, /μL	5346 ± 1690	5323 ± 1787	5326 ± 1633	0.981	0.870	0.978	0.841
Platelets, ×10^4^/μL	16.2 ± 5.6	16.5 ± 5.1	16.8 ± 5.5	0.289	0.844	0.129	0.554
Albumin, g/dL	3.6 ± 0.3	3.6 ± 0.4	3.7 ± 0.3	0.014	0.532	0.017	0.050
Ferritin, ng/mL	56.6 (22.0–124.8)	63.4 (29.9–180.2)	46.8 (23.0–98.0)	0.101	0.249	0.218	0.060
Transferrin saturation, %	21.9 ± 10.4	25.4 ± 15.9	24.1 ± 13.1	0.261	0.320	0.126	0.634
Serum iron, μg/dL	59.5 ± 28.6	72.4 ± 37.9	63.9 ± 27.4	0.016	0.012	0.025	0.118
C-reactive protein, mg/dL	0.14 (0.06–0.40)	0.14 (0.06–0.34)	0.1 (0.05–0.25)	0.009	0.949	0.005	0.144
Triglycerides, mg/dL	97 (70–133)	93 (69–122)	91 (65–133)	0.678	0.619	0.387	0.990
Total cholesterol, mg/dL	163 ± 34	165 ± 32	162 ± 35	0.884	0.786	0.790	0.654
LDL-C, mg/dL	87 ± 27	92 ± 21	89 ± 26	0.550	0.285	0.626	0.355
HDL-C, mg/dL	52 ± 17	54 ± 17	51 ± 16	0.855	0.594	0.936	0.585
Modality of dialysis, n (%)							
HD	166 (86%)	43 (98%)	656 (94%)	0.002	0.034	0.001	0.634
Hemodiafiltration	26 (14%)	1 (2%)	38 (5%)				
Hemofiltration	0 (0%)	0 (0%)	1 (0%)				
HD session length, hours/session	4.7 (0.6)	4.6 (0.6)	4.7 (0.6)	0.269	0.162	0.785	0.108
Weekly hours of HD, hours/week	14.1 (1.7)	13.7 (1.8)	14.1 (1.8)	0.448	0.233	0.974	0.211
Use of β2-MG apheresis column, n (%)	45 (23%)	4 (9%)	4 (1%)	<0.001	0.034	<0.001	<0.001
Use of HPM dialyzer, n/N (%)*	162/166 (98%)	43/43 (100%)	645/656 (98%)	0.544	0.304	0.529	0.392
Use of ultrapure dialysate, n (%)**	169 (88%)	41 (93%)	631 (91%)	0.423	0.324	0.253	0.592
Use of ESAs, n (%)	159 (83%)	32 (73%)	581 (84%)	0.178	0.125	0.796	0.063
Use of iron preparations, n (%)	69 (36%)	12 (27%)	210 (30%)	0.268	0.275	0.131	0.680
Use of benzodiazepines, n (%)	41 (21%)	5 (11%)	151 (22%)	0.263	0.131	0.912	0.102
Use of anti-depressants, n (%)	3 (2%)	2 (5%)	13 (2%)	0.420	0.215	0.777	0.222

Data are expressed as mean ± SD, median (interquartile range), or n (percentage) as appropriate.

Abbreviations: β2-MG, β2-microglobulin; BMI, body mass index; CGR, creatinine generation rate; DBP, diastolic blood pressure; DRA, dialysis-related amyloidosis; ESAs, erythropoiesis-stimulating agents; EQ-5D-3L, EuroQol 5-Dimensions 3-Levels Questionnaire; GNRI, geriatric nutritional risk index; HD, hemodialysis; HDL-C, high-density lipoprotein cholesterol; HPM, high-performance membrane; LDL-C, low-density lipoprotein cholesterol; nPCR, normalized protein catabolic rate, SBP, systolic blood pressure.

* Among only HD patients; HPM dialyzer was defined as types IV or V dialyzer.

** Ultrapure dialysate was defined as having levels of bacteria and endotoxin of <0.1 CFU/mL and <0.03 EU/mL, respectively.

We investigated the proportion of QOL decline, odds ratio, and change (EQ-5D-3L utility score after 2-year follow-up − EQ-5D-3L utility score at baseline). The proportion of patients with a decline in QOL was higher in Group 2 (i.e., patients newly complicated by DRA during the 2-year period) than in Group 3 (i.e., patients not complicated by DRA) (65.9% vs. 34.8%). Utility QOL scores were more likely to decline in Group 2, compared with Group 3, even after multivariable adjustment. QOL scores decline at the end of the 2 year-follow up was more likely to occur in Group 1 than in Group 3 (44,8% vs 34,8%). However, this difference became non-significant in multivariable analysis (**Table 2**).

**Table 2:** Odds ratio for decline in EQ-5D-3L utility score after 2 years of follow-up.

	Patients with Decline in QOL *n* (%)	Odds ratio (95%CI)	*P*
Univariable analysis			
Group 1	86 (44.8%)	1.52 (1.10 to 2.10)	0.012
Group 2	29 (65.9%)	3.62 (1.9 to 6.88)	<0.0001
Group 3	242 (34.8%)	Reference	
Multivariable analysis (Model 1)			
Group 1	86 (44.8%)	1.46 (0.94 to 2.25)	0.090
Group 2	29 (65.9%)	2.86 (1.36 to 6.00)	0.006
Group 3	242 (34.8%)	Reference	
Multivariable analysis (Model 2)			
Group 1	86 (44.8%)	1.45 (0.94 to 2.24)	0.095
Group 2	29 (65.9%)	2.75 (1.30 to 5.81)	0.008
Group 3	242 (34.8%)	Reference	
Multivariable analysis (Model 3)			
Group 1	86 (44.8%)	1.38 (0.93 to 2.05)	0.106
Group 2	29 (65.9%)	3.47 (1.76 to 6.85)	0.0003
Group 3	242 (34.8%)	Reference	

Model 1: Adjusted for age, age at hemodialysis onset, sex, diabetic nephropathy, dialysis vintage, history of myocardial infarction, ischemic stroke, intracerebral hemorrhage or amputation, predialysis body mass index, Kt/V, geriatric nutritional risk index, hemoglobin, C-reactive protein, use of benzodiazepine agents.

Model 2: Adjusted for covariates in Model 1 plus new-onset myocardial infarction, ischemic stroke, intracerebral hemorrhage or amputation during follow-up.

Model 3: Adjusted for all factors in Model 2 except for Kt/V and C-reactive protein.

Abbreviations: CI, confidence interval; EQ-5D-3L, EuroQol 5-Dimensions 3-Levels Questionnaire; QOL, quality of life.

Similarly, the change in utility score was significantly greater in Group 2 than in Group 3, and the change was comparable between Groups 1 and 3 (**Table 3**).

**Table 3:** Change in EQ-5D-3L utility score after 2 years of follow-up.

	Mean change (95% CI)	Difference (95% CI)	*P*
Univariable analysis			
Group 1	−0.03 (−0.06 to 0.00)	0.00 (−0.03 to 0.03)	0.826
Group 2	−0.13 (−0.18 to −0.08)	−0.10 (−0.15 to −0.04)	0.001
Group 3	−0.03 (−0.05 to −0.02)	Reference	
Multivariable analysis (Model 1)			
Group 1	−0.02 (−0.06 to 0.01)	−0.01 (−0.04 to 0.03)	0.701
Group 2	−0.08 (−0.14 to −0.02)	−0.06 (−0.13 to 0.00)	0.039
Group 3	−0.02 (−0.04 to 0.01)	Reference	
Multivariable analysis (Model 2)			
Group 1	−0.03 (−0.08 to 0.02)	−0.01 (−0.04 to 0.03)	0.710
Group 2	−0.09 (−0.16 to −0.02)	−0.06 (−0.12 to 0.00)	0.046
Group 3	−0.03 (−0.07 to 0.02)	Reference	
Multivariable analysis (Model 3)			
Group 1	−0.06 (−0.11 to −0.01)	−0.01 (−0.04 to 0.03)	0.637
Group 2	−0.15 (−0.22 to −0.09)	−0.10 (−0.16 to −0.04)	0.001
Group 3	−0.05 (−0.09 to −0.01)	Reference	

Model 1: Adjusted for age, age at hemodialysis onset, sex, diabetic nephropathy, dialysis vintage, history of myocardial infarction, ischemic stroke, intracerebral hemorrhage or amputation, predialysis body mass index, Kt/V, geriatric nutritional risk index, hemoglobin, C-reactive protein, use of benzodiazepine agents.

Model 2: Adjusted for covariates in Model 1 plus new-onset myocardial infarction, ischemic stroke, intracerebral hemorrhage or amputation during follow-up.

Model 3: Adjusted for all factors in Model 2 except for Kt/V and C-reactive protein.

Abbreviations: CI, confidence interval; EQ-5D-3L, EuroQol 5-Dimensions 3-Levels Questionnaire.

Group 1 was further divided into two groups in which β2-microglobulin-adsorbing Lixelle^®^ was used continuously throughout the 2-year follow-up period (45 patients, 23%) or not (147 patients, 77%); the proportion and odds ratio of decline and change in the utility score were investigated. The proportion of decline in the utility score was not different between patients with and without use of Lixelle^®^ (37.8% vs. 46.9%), nor was there any significant difference in the odds ratio of decline in the utility score (**Table 4**).

**Table 4:** Odds Ratio for a decline in EQ-5D-3L utility score after 2 years of follow-up according to use of β2-MG apheresis column.

	Number of Patients with decreased EQ-5D-3L utility score *n* (%)	Odds ratio (95% CI)	*P*
Crude analysis			
β2-MG apheresis column	17 (37.8%)	0.69 (0.35 to 1.36)	0.281
No β2-MG apheresis column	69 (46.9%)	Reference	
Multivariable analysis (Model 1)			
β2-MG apheresis column	17 (37.8%)	0.61 (0.27 to 1.4)	0.247
No β2-MG apheresis column	69 (46.9%)	Reference	
Multivariable analysis (Model 2)			
β2-MG apheresis column	17 (37.8%)	0.61 (0.27 to 1.4)	0.247
No β2-MG apheresis column	69 (46.9%)	Reference	
Multivariable analysis (Model 3)			
β2-MG apheresis column	17 (37.8%)	0.61 (0.28 to 1.31)	0.204
No β2-MG apheresis column	69 (46.9%)	Reference	

Model 1: Adjusted for age, age at hemodialysis onset, sex, diabetic nephropathy, dialysis vintage, history of myocardial infarction, ischemic stroke, intracerebral hemorrhage or amputation, predialysis body mass index, Kt/V, geriatric nutritional risk index, hemoglobin, C-reactive protein, use of benzodiazepine agents.

Model 2: Adjusted for covariates in Model 1 plus new-onset myocardial infarction, ischemic stroke, intracerebral hemorrhage or amputation during follow-up.

Model 3: Adjusted for all factors in Model 2 except for Kt/V and C-reactive protein.

Abbreviations: β2-MG, β2-microglobulin; CI, confidence interval; EQ-5D-3L, EuroQol 5-Dimensions 3-Levels Questionnaire.

Regarding the change in the utility score, the decline in the score tended to be smaller in patients with use of Lixelle^®^ compared to those without use of the column in the crude analysis and after Model 1 and 2 adjustments, although this was not significant; however, this was significantly smaller after the Model 3 adjustments (**Table 5**).

**Table 5:** Change in EQ-5D-3L utility score after 2 years of follow-up according to use of β2-microglobulin apheresis column.

	Mean change (95% CI)	Difference (95% CI)	*P*
Crude analysis			
β2-MG apheresis column	0.01 (−0.04 to 0.06)	0.05 (−0.01 to 0.11)	0.088
No β2-MG apheresis column	−0.04 (−0.07 to −0.01)	Reference	
Multivariable analysis (Model 1)			
β2-MG apheresis column	0.01 (−0.01 to 0.12)	0.07 (0.00 to 0.14)	0.062
No β2-MG apheresis column	−0.06 (−0.16 to 0.05)	Reference	
Multivariable analysis (Model 2)			
β2-MG apheresis column	0.09 (−0.07 to 0.26)	0.07 (0.00 to 0.14)	0.064
No β2-MG apheresis column	0.02 (−0.13 to 0.18)	Reference	
Multivariable analysis (Model 3)			
β2-MG apheresis column	−0.05 (−0.19 to 0.08)	0.08 (0.01 to 0.15)	0.017
No β2-MG apheresis column	−0.14 (−0.26 to −0.01)	Reference	

Model 1: Adjusted for age, age at hemodialysis onset, sex, diabetic nephropathy, dialysis vintage, history of myocardial infarction, ischemic stroke, intracerebral hemorrhage or amputation, predialysis body mass index, Kt/V, geriatric nutritional risk index, hemoglobin, C-reactive protein, use of benzodiazepine agents.

Model 2: Adjusted for covariates in Model 1 plus new-onset myocardial infarction, ischemic stroke, intracerebral hemorrhage or amputation during follow-up.

Model 3: Adjusted for all factors in Model 2 except for Kt/V and C-reactive protein.

Abbreviations: β2-MG, β2-microglobulin; CI, confidence interval; EQ-5D-3L, EuroQol 5-Dimensions 3-Levels Questionnaire.

We also examined the potential impact of β2-microglobulin apheresis column in the other 2 groups. Due to a very low proportion of use of β2-microglobulin apheresis column in Groups 2 and 3, there were no differences in the frequencies of decline in the utility score and changes in the utility score, although a slight tendency for the utility score to be maintained higher was observed in patients treated with the column compared to those untreated in both groups (**S4 and S5 Tables**).

## Discussion

We investigated the association between complication by DRA and decline in QOL both cross-sectionally and longitudinally using the EQ-5D-3L among 1,314 patients undergoing dialysis for more than 10 years. The utility score was significantly lower in patients clinically diagnosed with DRA compared with those without clinical DRA, indicating a lower QOL in patients with DRA. In the 2-year follow-up, it was clarified that the proportion of QOL decline was significantly higher in the patient group newly complicated by DRA than in the group not complicated by DRA. In the patient group already complicated by DRA at baseline, the decline in QOL tended to be ameliorated in patients with continuous use of Lixelle^®^ compared with those who did not use Lixelle^®^.

Several studies have been conducted evaluating QOL of patients undergoing dialysis, using the EQ-5D-3L utility score; a utility score in CKD stage G5 of 0.798 was reported in a single center study in Japan [21], and a wide distribution of scores (from 0.44 to 0.71) was reported in a systematic review conducted in 2012 [22]. In the present clinical study, we investigated the EQ-5D-3L utility score based on the presence or absence of clinically diagnosed DRA in patients undergoing dialysis for more than 10 years. This is the first report of the association of background and treatment method with utility score in patients undergoing dialysis investigated both cross-sectionally and longitudinally. The utility score distribution in the first survey was bimodal (**S1 Fig**) and a considerable number of patients reported no problems (utility score 1). A significant difference was noted in the utility score between patients with and without clinically diagnosed DRA (0.649 vs. 0.768), suggesting that the QOL of patients complicated by clinical DRA is low.

A previous study suggested that patients with DRA have chronic inflammation [23], which may cause decline in QOL; however, CRP elevation was not associated with utility score in multivariable analysis in our study. GNRI was positively associated with the utility score, suggesting the importance of nutritional management. The association of monotherapy with benzodiazepine and death was reported in the DOPPS survey [24]. Interestingly, a significant association of this drug with a decline in the utility score was evident in multivariable analysis in our clinical study.

We then investigated the associations of complications by the major symptoms of DRA and of treatment methods with QOL. Among the major symptoms of DRA, the prevalence of polyarthralgia accounted for the highest proportion, but the presence of an association between polyarthralgia and the utility score was not evaluated in this study. A significant association of arthralgia with gait disorder was clarified using multivariable analysis in the survey of the actual state performed by the Research Committee of Amyloidosis of the Ministry of Health, Labour and Welfare [5]. From this perspective, approaches to relieving polyarthralgia may be important to manage pathologies associated with the life prognosis of patients undergoing dialysis, such as impaired walking function, sarcopenia, and frailty. Administration of analgesics was not examined in this study but it has been described in the treatment guidelines for chronic pain published in 2018 in the Policy Research Project on Chronic Pain [25] from a Health and Labour Grant for administrative promotion. Those guidelines state that attention should be paid to the dose and long-term use of analgesics. Arthralgia should be controlled via dose reduction or withdrawal as much as possible in patients receiving dialysis. Of note, data showing that pain control and dose reduction with analgesics are possible with the use of a β2-microglobulin absorber, Lixelle^®^ has been described previously [26]. In addition, it has been reported that DRA may increase the risks of fall owing to joint contracture, enlargement of a bone cyst to a large size in the femoral neck, and femoral neck fracture [27]. Because improvement in ADL and inhibition of bone cyst advancement have been reported with treatment using Lixelle^®^ [28], a prospective intervention study on the improvement of walking function and reduction in fracture risk is anticipated.

A significant association was noted between the clinical diagnosis of DRA or the number of symptoms (three or more) and the EQ-5D-3L utility score. Thus, we investigated the treatment method based on the presence or absence of clinically diagnosed DRA in the first survey. In the group diagnosed with DRA, many patients underwent hemodiafiltration treatment (13.5% vs. 5.3%), implying that active β2-microglobulin removal was the therapeutic goal. Many patients concomitantly used Lixelle^®^ concomitantly in the group diagnosed with DRA (26.4% vs. 1.1%). Indeed, the serum β2-microglobulin level was 27.0 mg/L in Group 1 patients complicated by DRA at the baseline, which was lower than Group 3 patients not complicated by DRA (29.3 mg/L). However, the proportion of decline of the utility score and the odds ratio in Group 1 tended to be higher than those in Group 3, although not significantly, suggesting that under the treatment conditions of this study, once complicated by DRA, a decrease in patients’ utility score cannot be sufficiently inhibited compared with patients not complicated by DRA. In Group 2 patients newly complicated by DRA during the follow-up period, the number of major symptoms of DRA increased significantly and the proportion of utility score reduction was significantly higher than that in Group 3 patients, suggesting that the prevention of DRA development is critically important.

Serum β2-microglobulin level was higher in patients in Group 3 in spite of a shorter dialysis vintage and possibly preserved residual kidney function (RKF). However, those patients have not developed DRA. This seemingly contradictory finding may be explained as follows. Because all patients in this study had received hemodialysis for more than 10 years, RKF was thought to be almost completely abolished, suggesting that there might be no difference in RKF among the groups. Meanwhile, the proportion of patients treated with hemodiafiltration or β2-microglobulin apheresis column (Lixelle^®^) was higher in Group 1 compared to Group 3, whereas there was no difference in the proportion of those undergoing hemodialysis with high-performance membrane dialyzer or ultrapure dialysate. According to these findings, the higher serum β2-microglobulin levels in Group 3 patients might have resulted from lower proportion of patients treated with hemodiafiltration or β2-microglobulin apheresis column (Lixelle^®^), and the reason why those in Group 3 have not developed DRA might be due to a shorter dialysis vintage.

As Lixelle^®^ has been reported to be effective in improving DRA-induced arthralgia [26,28], reduction of the number of the major symptoms of DRA may be possible in Group 1, which may subsequently influence the utility score. Thus, we investigated the change in utility score among patients in Group 1 with and without continuous use of Lixelle^®^. In multivariable analysis (Model 3), the reduction in the EQ-5D-3L utility score was significantly smaller in the group with continuous use of Lixelle^®^. The health insurance coverage criteria of Lixelle^®^ in Japan requires: (1) surgical or biopsy confirmation of β2-microglobulin-induced amyloid deposition, (2) dialysis vintage 10 years or longer with a history of carpal tunnel release, and (3) the presence of bone cyst on imaging diagnosis. Because this was a survey of actual clinical practice, concomitant use of Lixelle^®^ was entrusted to the judgment of the attending physicians, and the use of Lixelle^®^ may have been biased in patients with aggravated DRA. Analysis by propensity score matching was attempted, but the sample size was insufficient for evaluation. It may be necessary to perform a longitudinal study using a larger sample size in a randomized controlled trial.

This study has several strengths, including a relatively large number of patients and prospectively collected baseline data. The baseline characteristics included various serum biochemical parameters, and possible confounding factors were vigorously adjusted using conventional statistical techniques. However, this study also had several limitations. First, we were unable to confirm causalities between DRA and decline in QOL and between use of Lixelle^®^ and maintenance of QOL owing to the observational nature of the study. Second, not all factors that may influence the EQ-5D-3L utility score were necessarily evaluated because the survey items were limited. Third, data on dialysis membrane and dialysate were recorded at the study entry; that is, biocompatible high-performance membrane and ultrapure dialysate have not been used all the time in patients with the longest hemodialysis vintage. Fourth, selection of the hemodialysis treatment method was entrusted to the clinicians. That is, DRA may have already been aggravated when the health insurance coverage criteria for Lixelle^®^ in Japan were followed; as a result, the background of patients treated with Lixelle^®^ may have differed from that of patients not using Lixelle^®^. We believe that the importance of this study is not greatly impaired by these limitations because of the study strengths.

## Conclusion

According to the findings of the present study, we can conclude that complication by DRA in patients undergoing hemodialysis is associated with a decline in QOL.

## Supporting information

S1 Fig Distribution of EQ-5D-3L utility scores in all patients (*N* = 1,314). Abbreviation: EQ-5D-3L, EuroQol 5-Dimensions 3-Levels Questionnaire.
(DOCX)

S1 Table Distribution of the answers to the EQ-5D-3L dimensions. (DOCX)

S2 Table Baseline characteristics and laboratory data. (DOCX)

S3 Table Multivariable association of DRA with EQ-5D-3L utility scores. (DOCX)

S4 Table Proportion of decline in EQ-5D-3L utility score after 2 years of follow-up according to use of β2-MG apheresis column. (DOCX)

S5 Table Mean change (95% confidence interval) in EQ-5D-3L utility score after 2 years of follow-up according to use of β2-MG apheresis column. (DOCX)
