# Supplementary material for: Association of dialysis-related amyloidosis with lower quality of life in patients undergoing hemodialysis for more than 10 years: The Kyushu Dialysis-Related Amyloidosis Study
Source: PLoS One. 2021 Aug 24;16(8):e0256421. doi: 10.1371/journal.pone.0256421 (PMC8384206; doi:10.1371/journal.pone.0256421)
Supplement: S1 Fig — Abbreviation: EQ-5D-3L, EuroQol 5-Dimensions 3-Levels Questionnaire. (DOCX) [file pone.0256421.s001.docx]

**S1 Fig. Distribution of EQ-5D-3L Utility Scores in All Patients (*N* = 1,314).**

**3**


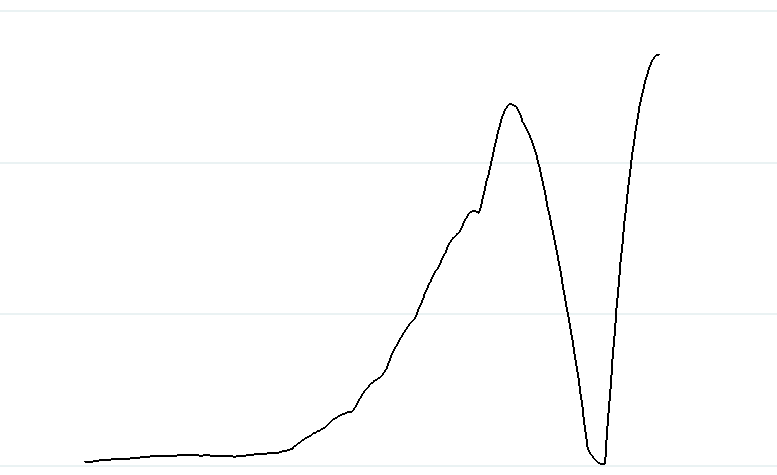


**Figure 3**

**2**

**Density**

**1**

**0**

**1.0**

**0.8**

**EQ-5D-3L Utility Score**

**0.6**

**0.4**

**0.2**

**0**

**−0.2**

Abbreviation: EQ-5D-3L, EuroQol 5-Dimensions 3-Levels Questionnaire.
