# Supplementary material for: Association of dialysis-related amyloidosis with lower quality of life in patients undergoing hemodialysis for more than 10 years: The Kyushu Dialysis-Related Amyloidosis Study
Source: PLoS One. 2021 Aug 24;16(8):e0256421. doi: 10.1371/journal.pone.0256421 (PMC8384206; doi:10.1371/journal.pone.0256421)
Supplement: S1 Table — (DOCX) [file pone.0256421.s002.docx]

**S1 Table. Distribution of the answers to the EQ-5D-3L dimensions**

| **Health domains** | **Options** | **%** |
| --- | --- | --- |
| Mobility | 1. I have no problems in walking about 2. I have some problems in walking about 3. I am confined to bed | 64  34  2 |
| Self-care | 1. I have no problems with self-care 2. I have some problems washing or dressing myself 3. I am unable to wash or dress myself | 84  14  3 |
| Usual activities | 1. I have no problems with performing my usual activities 2. I have some problems with performing my usual activities 3. I am unable to perform my usual activities | 67  29  4 |
| Pain/Discomfort | 1. I have no pain or discomfort 2. I have moderate pain or discomfort 3. I have extreme pain or discomfort | 45  47  8 |
| Anxiety/Depression | 1. I am not anxious or depressed 2. I am moderately anxious or depressed 3. I am extremely anxious or depressed | 78  20  2 |

Abbreviations: EQ-5D-3L, EuroQol 5-Dimensions 3-Levels Questionnaire.
