# Supplementary material for: Association of dialysis-related amyloidosis with lower quality of life in patients undergoing hemodialysis for more than 10 years: The Kyushu Dialysis-Related Amyloidosis Study
Source: PLoS One. 2021 Aug 24;16(8):e0256421. doi: 10.1371/journal.pone.0256421 (PMC8384206; doi:10.1371/journal.pone.0256421)
Supplement: S2 Table — (DOCX) [file pone.0256421.s003.docx]

**S2 Table. Baseline Characteristics and Laboratory Data**

| Variables (*N* = 1,314) |  |
| --- | --- |
| Age, years | 64.7 ± 10.5 |
| Age at HD initiation, years | 45.8 ± 13.0 |
| Male, n (%) | 715 (54%) |
| Dialysis vintage, years | 17 (13–24) |
| Previous history, n (%) |  |
| Myocardial infarction | 79 (6%) |
| Brain hemorrhage | 61 (5%) |
| Brain infarction | 154 (12%) |
| Amputation of extremities | 17 (1%) |
| Body height, cm | 159.0 ± 9.3 |
| Pre-dialysis body weight, kg | 55.2 ± 11.1 |
| Post-dialysis body weight, kg | 52.9 ± 10.6 |
| Pre-dialysis SBP, mmHg | 145.4 ± 22.6 |
| Pre-dialysis DBP, mmHg | 75.7 ± 13.5 |
| Cardiothoracic ratio, % | 50.6 ± 5.3 |
| EQ-5D-3L utility score | 0.768 (0.649–1.000) |
| Kt/V | 1.75 ± 0.46 |
| CGR, % | 108.7 ± 33.4 |
| nPCR, g/kg/day | 0.94 ± 0.20 |
| GNRI | 99.1 ± 10.1 |
| β2-MG, mg/L | 28.8 ± 6.5 |
| Hemoglobin | 10.9 ± 1.2 |
| White blood cells, /µL | 5334 ± 1695 |
| Platelets, × 10^4^/µL | 16.4 ± 5.6 |
| Albumin, g/dL | 3.6 ± 0.4 |
| Ferritin, ng/mL | 51.8 (24.9–112.2) |
| Transferrin saturation, % | 23.9 ± 12.4 |
| Serum iron, µg/dL | 62.6 ± 28.3 |
| C-reactive protein, mg/dL | 0.11 (0.05–0.32) |
| Triglycerides, mg/dL | 91 (64–130) |
| Total cholesterol, mg/dL | 160 ± 35 |
| LDL-C, mg/dL | 87 ± 26 |
| HDL-C, mg/dL | 51 ± 16 |
| Modality of dialysis, n (%) |  |
| HD | 1201 (91%) |
| Hemodiafiltration | 110 (8%) |
| Hemofiltration | 3 (0.2%) |
| HD session length, hours/session | 4.6 (0.6) |
| Weekly hours of HD, hours/week | 13.9 (1.8) |
| Use of β2-MG apheresis column, n (%) | 83 (6%) |
| Use of HPM dialyzer, n/N (%)* | 1174/1201 (98%) |
| Use of ultrapure dialysate, n (%)** | 1207 (92%) |

Data are expressed as mean ± SD, median (interquartile range), or n (percentage) as appropriate.

Abbreviations: β2-MG, β2-microglobulin; BMI, body mass index; CGR, creatinine generation rate; DBP, diastolic blood pressure; DRA, dialysis-related amyloidosis; ESAs, erythropoiesis-stimulating agents; EQ-5D-3L, EuroQol 5-Dimensions 3-Levels Questionnaire; GNRI, geriatric nutritional risk index; HD, hemodialysis; HDL-C, high-density lipoprotein cholesterol; HPM, high-performance membrane; LDL-C, low-density lipoprotein cholesterol; nPCR, normalized protein catabolic rate, SBP, systolic blood pressure.

* Among only HD patients; HPM dialyzer was defined as types IV or V dialyzer.

** Ultrapure dialysate was defined as having levels of bacteria and endotoxin of <0.1 CFU/mL and <0.03 EU/mL, respectively.
