# Supplementary material for: Association of dialysis-related amyloidosis with lower quality of life in patients undergoing hemodialysis for more than 10 years: The Kyushu Dialysis-Related Amyloidosis Study
Source: PLoS One. 2021 Aug 24;16(8):e0256421. doi: 10.1371/journal.pone.0256421 (PMC8384206; doi:10.1371/journal.pone.0256421)
Supplement: S3 Table — (DOCX) [file pone.0256421.s004.docx]

**S3 Table. Multivariable Association of DRA with EQ-5D-3L Utility Scores**

|  | **OR** | **95% CI** | ***P*** | **OR** | **95% CI** | ***P*** |
| --- | --- | --- | --- | --- | --- | --- |
| Age, years | 0.95 | 0.94–0.96 | <0.001 | 0.95 | 0.94–0.97 | <0.001 |
| Male | 1.63 | 1.21–2.20 | 0.001 | 1.47 | 1.09–2.00 | 0.012 |
| Diabetic kidney disease | 0.33 | 0.21–0.50 | <0.001 | 0.31 | 0.20–0.48 | <0.001 |
| Dialysis vintage, years | 0.94 | 0.92–0.96 | <0.001 | 0.96 | 0.93–0.98 | <0.001 |
| Previous history * | 0.47 | 0.34–0.66 | <0.001 | 0.46 | 0.33–0.65 | <0.001 |
| Body mass index | 0.91 | 0.85–0.97 | 0.005 | 0.90 | 0.84–0.97 | 0.004 |
| Kt/V | 1.25 | 0.90–1.75 | 0.186 | 1.11 | 0.79–1.55 | 0.541 |
| GNRI | 1.06 | 1.03–1.09 | <0.001 | 1.06 | 1.03–1.09 | <0.001 |
| Hemoglobin, g/dL | 1.00 | 0.88–1.13 | 0.979 | 0.97 | 0.86–1.10 | 0.682 |
| C-reactive protein, mg/dL | 1.06 | 0.92–1.23 | 0.413 | 1.08 | 0.93–1.25 | 0.312 |
| Use of benzodiazepine drugs | 0.56 | 0.41–0.78 | <0.001 | 0.57 | 0.41–0.79 | <0.001 |
| Presence of DRA | 0.42 | 0.28–0.61 | <0.001 | − | − | − |
| Number of main symptoms of DRA  One symptom  Two symptoms  ≥3 symptoms | − | − | − | 0.98  0.89  0.47 | 0.75–1.28  0.64–1.25  0.32–0.69 | 0.884  0.514  <0.001 |

*Previous history of acute myocardial infarction, brain hemorrhage, brain infarction, or amputation of extremities.

Abbreviations: CI, confidence interval; DRA, dialysis-related amyloidosis; EQ-5D-3L, EuroQol 5-Dimensions 3-Levels Questionnaire; GNRI, geriatric nutritional risk index; OR, odds ratio.
