# Supplementary material for: Association of dialysis-related amyloidosis with lower quality of life in patients undergoing hemodialysis for more than 10 years: The Kyushu Dialysis-Related Amyloidosis Study
Source: PLoS One. 2021 Aug 24;16(8):e0256421. doi: 10.1371/journal.pone.0256421 (PMC8384206; doi:10.1371/journal.pone.0256421)
Supplement: S4 Table — (DOCX) [file pone.0256421.s005.docx]

| **S4 Table. Frequency of Decline in EQ-5D-3L Utility Score after 2 Years of Follow-Up According to Use of β2-MG Apheresis Column** | | | |
| --- | --- | --- | --- |
| n/n (%) | Group 1 (*n* = 192) | Group 2 (*n* = 44) | Group 3 (*n* = 695) |
| β2-MG apheresis column | 17/45 (37.8%) | 2/2 (50.0%) | 1/4 (25.0%) |
| No β2-MG apheresis column | 69/147 (46.9%) | 27/40 (67.5%) | 241/691 (34.9%) |
| *P* | 0.281 | 0.481 | 0.679 |

Abbreviations: β2-MG, β2-microglobulin; EQ-5D-3L, EuroQol 5-Dimensions 3-Levels Questionnaire.
