# Supplementary material for: Association of dialysis-related amyloidosis with lower quality of life in patients undergoing hemodialysis for more than 10 years: The Kyushu Dialysis-Related Amyloidosis Study
Source: PLoS One. 2021 Aug 24;16(8):e0256421. doi: 10.1371/journal.pone.0256421 (PMC8384206; doi:10.1371/journal.pone.0256421)
Supplement: S5 Table — (DOCX) [file pone.0256421.s006.docx]

| **S5 Table. Mean Change (95% Confidence Interval) in EQ-5D-3L Utility Score after 2 Years of Follow-Up According to Use of β2-MG Apheresis Column** | | | |
| --- | --- | --- | --- |
|  | Group 1 (*n* = 192) | Group 2 (*n* = 44) | Group 3 (*n* = 695) |
| β2-MG apheresis column | 0.01 (−0.04 to 0.06) | −0.09 (−0.32 to 0.14) | 0.01 (−0.07 to 0.09) |
| No β2-MG apheresis column | −0.04 (−0.07 to −0.01) | −0.13 (−0.19 to −0.08) | −0.03 (−0.05 to −0.02) |
| *P* | 0.088 | 0.613 | 0.640 |

Abbreviations: β2-MG, β2-microglobulin; EQ-5D-3L, EuroQol 5-Dimensions 3-Levels Questionnaire.
